# Reduction of p11 in dorsal raphe nucleus serotonergic neurons mediates depression-like behaviors

**DOI:** 10.1038/s41398-023-02664-3

**Published:** 2023-11-22

**Authors:** Wei Li, Zuqi Shen, Xuan Yin, Weiqi Chang, Xiaorong Chen, Jin Yu, Shifen Xu

**Affiliations:** 1 Department of Acupuncture and Moxibustion, Shanghai Municipal Hospital of Traditional Chinese Medicine, Shanghai University of Traditional Chinese Medicine, Shanghai, 200071 China; 2 Department of Integrative Medicine and Neurobiology, School of Basic Medical Sciences, Shanghai Medical College, Fudan University, Shanghai, 200032 China; 3 Shanghai Key Laboratory of Acupuncture Mechanism and Acupoint Function, Fudan University, Shanghai, 200433 China; 4 Department of Physiology, Laboratory of Neurodegenerative diseases, Changzhi Medical College, Changzhi, Shanxi 046000 China

**Keywords:** Depression, Molecular neuroscience

## Abstract

The pathology of depression is related to the imbalance of various neurotransmitters. The dorsal raphe nucleus (DRN), the main brain region producing 5-HT, is crucially involved in the pathophysiology of depression. It contains several neuron types, in which GABAergic neurons are activated by stimuli associated with negative experiences and 5-HT neurons are activated by reward signals. However, little is known about its underlying molecular mechanisms. Here, we found that p11, a multifunctional protein associated with depression, was down-regulated by chronic social defeat stress in 5-HT^DRN^ neurons. Knockdown of p11 in DRN induced depression-like behaviors, while its overexpression in 5-HT^DRN^ neurons alleviated depression-like behavior caused by chronic social defeat stress. Further, p11 regulates membrane trafficking of glutamate receptors in 5-HT^DRN^ neurons, suggesting a possible molecular mechanism underlying the participation of p11 in the pathological process of depression. This may facilitate the understanding of the molecular and cellular basis of depression.

## Introduction

Major depressive disorder (MDD) is a common mental illness that affects about 280 million people worldwide [1], Particularly due to the COVID-19 pandemic, initial estimates showed a 28% increase in MDD globally [2]. The prevalence and severity of MDD make it a global area of urgent concern, and more clinical and scientific research is needed to develop its diagnosis and treatment. Recently, the role of p11 (S100A10) in MDD has gained research traction. Patients with MDD and post-traumatic stress disorder show decreased levels of p11 in the brain [3–6]. Svenningsson et al. have shown that constitutive knockout of p11 in mice (p11-KO) results in a depression-like phenotype [7]. Furthermore, this effect has been replicated in the region- and cell-specific knockouts, such as in cholinergic interneurons of the nucleus accumbens (NAc) [3, 8]. Interestingly, p11 in dopamine D2 receptor-containing lateral habenula (LHb) neurons exhibits opposite effects on depression. On the contrary, overexpression of p11 in these neurons induces depression-like behaviors [9]. Taken together, accumulating evidence [3, 8–16] suggests that multiple brain regions are involved in the etiology of MDD and underlie region specificity of the p11-dependent mechanism of action and antidepressant responses. Moreover, A recent study of cell- and region-specific expression patterns of the depression-related protein, p11, in the brain has shown its widespread and differential expression in multiple brain regions [17]. Except for the cortex, NAc, and hippocampus, p11 is also highly expressed in the ventral, dorsal, and lateral parts of the dorsal raphe nuclei (DRN), as well as in dorsal raphe caudal (DRC) and interfascicular nuclei (DRI). The group speculated that p11 might be expressed in a subpopulation of neurons within the dorsal raphe nuclei (DRN) but they did not give more cues about their hypothesis and did not further clarify the function of p11 in DRN.

Accumulating data have shown that p11 plays an important role in the trafficking of transmembrane proteins. Hence, the regional specificity of p11’s effect may be related to the unique combination of different neurotransmitter receptors and ion channels expressed in neuronal subtypes found in different brain regions. Especially in the studies related to the pathophysiology and therapy of depression, p11 stabilizes 5-HT1BR, 5-HT1DR, 5-HT4R, and mGluR5 on the cell membrane, thus regulating the cellular response to the corresponding neurotransmitters in different brain regions [7, 9, 12, 18–21]. However, different neurons express varying patterns of neurotransmitter receptors, hence the behavioral outcome of perturbed p11-dependant signaling depends on the specific type of neurotransmitter receptors expressed in specific neurons of specific brain regions.

Moreover, DRN is best known as the origin of extensive serotonergic projections to the forebrain [22–26], whose involvement in reward processing has been extensively demonstrated. Apart from 5-HT neurons, the DRN contains a large proportion (estimated 50–75%) of non-serotonergic neurons comprised primarily of GABA- and glutamate-producing neurons [23, 27]. Recent trans-synaptic tracing studies have revealed that DRN GABA neurons share a largely similar input pattern with 5-HT neurons [28, 29]. As the major interneurons in the DRN, GABA neurons mono-synaptically inhibit 5-HT neurons [30] and play a key role in neuroplastic processes underlying the development of social avoidance [31]. Therefore, p11 might mediate opposite actions on depression-like behaviors if it is predominantly expressed on 5-HT or GABA neurons. According to the abovementioned studies, the present work was designed to investigate whether p11 has cell-specific expression in DRN and assess the involvement of p11 in DRN in chronic social defeat stress (CSDS)-induced depression-like phenotypes.

## Materials and methods

### Experimental animals

For all experiments, male C57BL/6J mice (7–8 weeks, from Charles River, China) and single-housed male CD-1 mice (7-month from Charles River, China). B6.Cg-Tg(Fev-cre)1Esd/J(Stock No:012712 7-8 weeks, from Jackson lab, USA). All mice were group-housed in a controlled environment (23 ± 2 °C on a 12 h light/dark cycle) with food and water available ad libitum. Mice were singly housed 1 week before resident-intruder testing and 24 h before social interaction testing. Behavioral assessments and tissue collections were performed during the animals’ light phase. This study was carried out by the National Institute of Health Guide for the Care and Use of Laboratory Animals. All the animal experiments were approved by the Animal Experiment Ethics Committee of Shanghai University of Traditional Chinese Medicine (PZSHUTCM200821009). According to the pre-experimental results, we determined the sample size of each group and numbered each mouse to make sure that they were randomly assigned to different groups by random number table.

### Chronic social defeat stress

The CSDS procedure was performed as previously described. C57BL/6J mice were individually introduced to the home cage of an unfamiliar aggressive CD-1 resident mouse for 5–10 min and exposed to physical defeat. After the initial defeat, the C57BL/6J mice were housed together with the CD-1 mouse, but separated by a perforated plastic divider to allow for visual, olfactory, and auditory contact for the remainder of the 24 h. This social defeat was repeated for 10 consecutive days, with each day involving exposure to a different CD-1 mouse. Control mice were pair-housed in defeat boxes, separated by the perforated plastic divider for the duration of the 10 days [32].

### Behavioral assessments

All behavioral tests were performed during the light cycle in a dedicated sound-proof behavioral facility by experimenters blind to treatment and genotype information. Mice were brought to the testing room 30 min before the start of each behavioral test and remained in the same room throughout the test.

#### Social interaction test

The mice were individually placed in a clean open field apparatus (50 cm × 50 cm × 50 cm) for the test. In the first session lasting 2.5 min (“no target”), each C57BL/6J mouse had unrestricted exploration of the arena with an empty cage (10 cm × 10 cm × 42 cm), allowing for visual, olfactory, and auditory contact. In the second session also lasting 2.5 min (“target”), the empty cage was replaced by a cage containing an unfamiliar aggressive CD1 mouse, specifically for each defeated C57BL/6J mouse. The amount of time spent in the interaction zone (10 cm around the cage) by the C57BL/6J mouse was measured in both sessions. The social interaction ratio (SIR) was then calculated as the ratio of time spent in the interaction zone with a target (CD1 mouse) to the time spent in the interaction zone with no target [32]. Consistent with previous studies [33], mice were classified as “resilient” if they spent more time in the interaction zone during the session with a target compared to the session with no target (SIR ≥ 1), while mice with SIR < 1 were classified as “susceptible”.

#### Sucrose preference test

Mice were presented with two water bottles. After habituation for 1 day, mice were given a free choice between two bottles, containing tap water or 2% sucrose solution. To prevent a possible effect of drinking behavior, the left/right location of the bottles was switched twice every day. After being deprived of food and water for one day. The consumption of water and sucrose solution was measured daily for 6 h by weighing the bottles. The sucrose preference was calculated as the ratio of consumed sucrose solution to consumed water.

#### Open field test

The apparatus for the open field test was a box (50 cm × 50 cm × 50 cm). The central area was defined as a central 20 cm × 20 cm square, and the other region was defined as the peripheral area. Mice were placed in the center of the open field box, and the distance traveled and the time spent in the center of the open field box in 30 min were recorded. Entry into the central area was defined as the placement of all four paws in the central zone of the open field apparatus. We obtained information about the duration spent and distance in the center zone.

#### Forced swimming test

Mice were placed individually in transparent pyrex cylinder (25 cm in height, 10 cm in diameter), filled with water at 25 °C to a height of 15 cm. Immobility time (sec) was recorded with a camera for 5 min. The immobility time was defined as the time when the mouse stopped struggling in the water, floated, or had only minor limb movements to keep its head above the water.

#### mRNA sequencing

Total RNA was extracted using the TRIzol reagent (Invitrogen, CA, USA) according to the manufacturer’s protocol. RNA purity and quantification were evaluated using the NanoDrop 2000 spectrophotometer (Thermo Scientific, USA). RNA integrity was assessed using the Agilent 2100 Bioanalyzer (Agilent Technologies, Santa Clara, CA, USA). Then the libraries were constructed using VAHTS Universal V6 RNA-seq Library Prep Kit according to the manufacturer’s instructions. The transcriptome sequencing and analysis were conducted by OE Biotech Co., Ltd. (Shanghai, China).

The libraries were sequenced on a illumina Novaseq 6000 platform and 150 bp paired-end reads were generated. Raw reads of fastq format were firstly processed using fastp and the low-quality reads were removed to obtain the clean reads. The clean reads were mapped to the reference genome using HISAT2. FPKM of each gene was calculated and the read counts of each gene were obtained by HTSeq-count. Differential expression analysis was performed using the DESeq2. *p*-value < 0.001 was set as the threshold for significantly differential expression genes (DEGs). Hierarchical cluster analysis of DEGs was performed using R (v 3.2.0) to demonstrate the expression pattern of genes in different groups and samples. Based on the hypergeometric distribution, GO, and KEGG pathway analysis of DEGs was performed to screen the significantly enriched term using R (v 3.2.0). All data has been submitted to the GEO(GSE247136).

#### Immunohistochemistry

Brains were perfused transcardially with cold PBS, followed by 4% paraformaldehyde (PFA), and postfixed in the same solution overnight at 4 °C. The brains were coronally cut into 30 µm-thick sections with a vibratome (VT 1000S, Leica). After blocking for 1.5 h, sections were incubated with the primary antibodies diluted in the blocking buffer. The immunohistochemistry was done using the following antibodies: anti-p11 (goat polyclonal, 1:500, R&D systems, #AF2377), anti-CaMKII (rabbit polyclonal, 1:500, CST, #50049), anti-Tph2 (rabbit polyclonal, 1:500, NOVUS, #NB100-74555), anti-NMDAR2A/ GRIN2A (rabbit polyclonal, 1:500, Protientech, #28525-1-AP) and anti-NMDAR2B/ GRIN2B (rabbit polyclonal, 1:500, Protientech, #21920-1-AP) After 24 h incubation, sections were washed, and incubated with Alexa-fluor-conjugated secondary antibodies (1:500, Invitrogen, Carlsbad, CA, USA). Slices were washed three times in PBST for 15 min each and mounted with Vectashield mounting medium with DAPI (Vector Laboratories, Burlingame, CA, USA) onto microscope slides. All the sections were examined under a Zeiss LSM710 confocal microscope or wide-field fluorescence microscope (Zeiss, Jena, Germany). All histology findings were confirmed in at least five different animals.

#### Virus construction

The target gene is mouse S100A10 (NM_009112). The target sequence of S100A10 shRNA was GCTTACGTTTCACAGGTTT, and the target was constructed into the pAAV-Syn-MCS-egfp-3Flag vector with the help of Micro30 structure, which contained the mature neuron promoter of SYN. After the construction of pAAV-Syn-MCS-egfp-3Flag-micro30-shRNA(S100A10), pAAV-Syn-MCS-egfp-3Flag was used as a control. The constructed plasmid, pHelper, and pAAV-RC were co-transfected into 293 T cells to package the AAV virus of serotype 9. The virus was designed by Shanghai Heyuan Biotech.

#### Stereotaxic surgery

All stereotaxic injections were carried out on a stereotaxic frame for the mouse with a motorized nanoinjector (Leica, Buffalo Grove, IL, USA). Ten-week-old male mice were anesthetized with isoflurane gas hemp. Injected with AAV-hSyn-EGFP-2A-sh. RNA, AAV-hSyn-EGFP-2A-sh.RNA-s100a10, AAV-EF1a-DIO-mCherry-2A-S100a10-3FLAG, AAV-EF1a-DIO-mCherry into the DRN (AP, −3.3 mm, ML, ±0.05 mm, DV, −4.4 mm from bregma). The total injection volume was 0.5 µl. All injections were performed at a rate of 0.15 µl/ min using Hamilton syringes (33 gauge, Reno, NV, USA) and the needle was kept in place for an additional 5 min. After 3 weeks of injection, depression-like behavioral tests were performed.

#### Pull-down and target validation

To identify the interacting cellular protein targets of p11, we designed pull-down experiments which were followed by LC-MS/MS and immunoblotting assays. Protein extracted from DRN were incubated with primary antibodies or control IgG in a rotating incubator overnight at 4 °C, followed by incubation with protein A/G magnetic beads (Bimake) for another 2 h. The immunoprecipitates were washed 3 times with lysis buffer.

After the precipitated proteins were subsequently centrifuged, the pellet was resuspended in PBS containing 1% SDS. Upon incubation with Streptavidin Mag Sepharose ™ pre-balanced with Binding buffer (50 mM Tris-HCl, 150 mM NaCl, pH 7.5) by using the MagRack 6 for 2 h at room temperature, the sepharose was washed with PBS containing 1% SDS (3 × 1 mL) and Washing buffer (Binding buffer, containing 2 M urea, pH 7.5) (3 × 1 mL). Finally, the enriched proteins were eluted in Novex™ Tris-Glycine SDS Sample Buffer (2×) at 95 °C for 10 min and separated by SDS-PAGE (10%). Control pull-down experiments using the negative probe (NP) were conducted simultaneously in vitro or situ as a negative control. For LC-MS/MS protein identification, silver staining by using Silver Xpress™ Silver Staining Kit of SDS-PAGE gels was conducted following the manufacturer’s recommendations. Each lane was cut into gel slices and subjected to LC-MS/MS analysis at Shanghai Luming Biological Technology Co., Ltd (Shanghai, China). Briefly, the peptide samples were re-dissolved in Nano-HPLC buffer A and then separated by Nano-HPLC liquid phase system based on EASY-nL C1000. Then the samples were loaded by an automatic sampler and absorbed on the Trap column, and then the samples were separated by the Analysis column (75 μm × 150 mm, RP-C18, Thermo Scientific) at a flow rate of 300 nL/min. The enzymatic hydrolysis products were separated by capillary high-performance liquid chromatography and analyzed on a mass spectrometer. Finally, the target proteins of 10i were identified by comparison with the Uniprot-Mus musculus database using the Mascot2.3 software. Then immunoblotting experiments were conducted as described below using the corresponding antibodies.

#### Western blot analysis

Harvested for separation of total cellular components into different fractions with Minute Plasma Membrane Protein Isolation and Cell Fractionation Kit (Invent Biotechnologies) according to the manufacturer’s instructions. The supernatant was collected, and the protein concentration of each sample was determined by the bicinchoninic acid (BCA) method (Thermo Fisher, USA). The same total protein levels were separated on 12 to 15% SDS-PAGE for gel electrophoresis and electrotransferred to PVDF membranes (Millipore, USA). Blots were then blocked with a blocking buffer for 2 h at room temperature (RT). After washing, PVDF membranes containing proteins were incubated with primary antibodies overnight at 4 °C. After three washes with TBST for 1 h, PVDF membranes were incubated with secondary antibodies for 2 h at RT washed three times with TBST for 10 min each. The ECL reagent (Millipore, USA) observed the immune response. Tubulin antibodies (Rabbit mAb, 1:500, Proteintech, *#*1124-1-AP) and PSD95 (rabbit polyclonal, 1:500, Protientech, *#*20665-1-AP) were used as internal control probes. anti-NMDAR2A/GRIN2A (rabbit polyclonal, 1:500, Protientech, *#*28525-1-AP) and anti-NMDAR2B/GRIN2B (rabbit polyclonal, 1:500, Protientech *#*21920-1-AP) was probed for these proteins. Densitometric analysis of immunoblots was performed using Image J analysis software.

#### Transmission electron microscopy and immunogold staining

Brain specimens for transmission electron microscopy and immunogold staining. Briefly, ultrathin sections (100 nm) of kidneys fixed with 0.1% osmium tetroxide postfixed paraformaldehyde-glutaraldehyde were embedded in epoxy resin and mounted on copper grids. Sections were stained with 2% aqueous uranyl acetate and Raynor’s lead citrate and examined by transmission electron microscopy using an EM208s microscope (Philips Eindhoven). Brains were fixed with para formalin-glutaraldehyde for immunogold staining, embedded in LR white resin (medium grade), and ultrathin sections mounted on nickel mesh. Sections were washed with 0.05 M glycine in PBS and blocked with AURION donkey serum blocking solution. Sections were washed twice with PBS containing BSA and sodium azide (PBS-BSA-nan3 buffer), incubated with goat anti-p11 antibody (1:50 diluted in PBS-BSA-nan3 buffer) at 4 °C overnight, and then rinsed with PBS-BSA-nan3 buffer. The cells were incubated with 10 nm gold-labeled donkey anti-goat IgG secondary antibody. Sections were counterstained with uranyl acetate and lead citrate and examined by transmission electron microscopy.

#### Statistical analysis

If the data followed normal distribution and homogeneity of variance, *t* test analysis of variance was used for comparison between two groups, and the two-way analysis of variance (ANOVA) followed by the Tukey HSD was used for repeated measures comparisons of two groups. If normal distribution or homogeneity of variance was not followed, non-parametric test was used. No blinding method was used in the study. The data are presented as the standard error (SEM). All statistical analyses were performed and all graphs were plotted using the GraphPad Prism 6.01 (GraphPad Software Inc., San Diego, CA, USA). We used G. power to do Bonferroni tests and Tukey tests for post hoc analysis. The statistical significance level was set at *p* < 0.05.

## Results

### Effects of CSDS on p11 expression in DRN

**Fig. 1:**
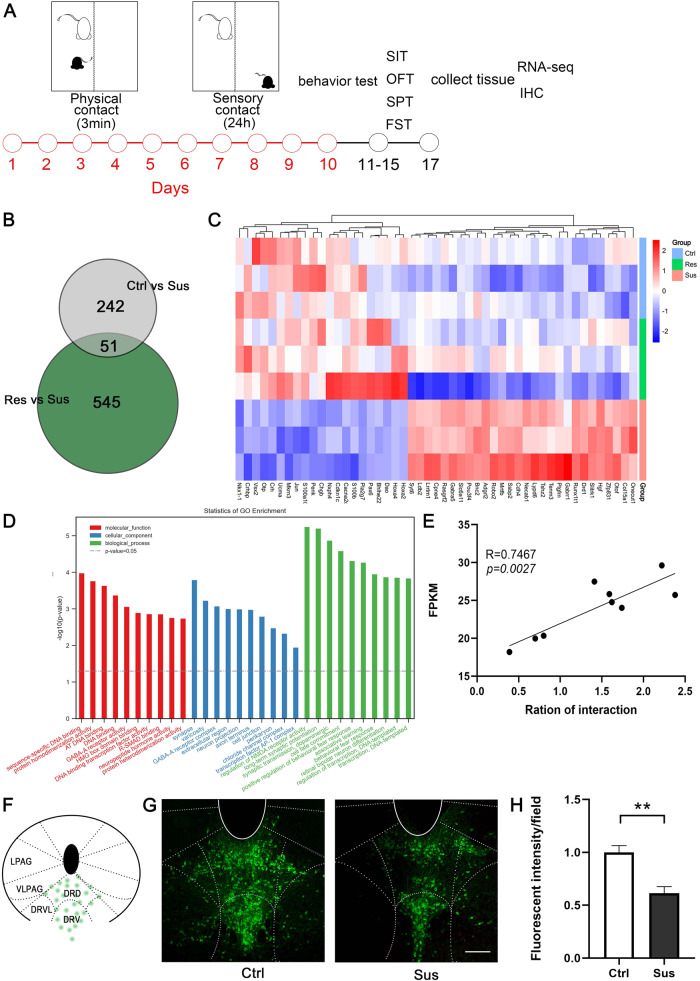
Effects of CSDS on p11 expression in DRN. **A** Schematic paradigm of CSDS and experimental timeline for social interaction test (SIT), open field test (OFT), sucrose preference test (SPT) and forced swimming test (FST). **B** Venn diagram of differentially expressed genes from Ctrl, Sus and Res. **C** Heatmap showing relative expression levels of 51 differentially expressed genes (DEGs, adjusted *p* < 0.1 by Deseq2) in the DRN comparing Ctrl with Sus groups. Color scale bar values represent standardized rlog-transformed values across samples. **D** GO pathway of 51 differentially expressed genes revealed by gene set enrichment analysis (GSEA). GO-BP: gene ontology – biological process, GO-CC: gene ontology – cellular component, GO-MF: gene ontology – molecular function. **E** Correlation analysis of p11 mRNA expression level and social index (*n* = 9 *p* = 0.0027 R = 0.746). **F** Schematic representation of p11 distribution in DRN. **G** Immunofluorescence staining of p11, Ctrl-left, Sus-right, scale bar 250 μM. **H** Comparison of fluorescence intensity of p11 expression in DRN of Sus and Ctrl (Ctrl *n* = 5, Sus *n* = 5, bars represent mean ± s.e.m. Two-tailed unpaired Students *t* test, *p* = 0.0024 *t* = 4.366). In all panels, *p < 0.05, **p < 0.01, ***p < 0.001, ns *p* > 0.05.

We found behavioral differences in the mice after CSDS modeling. Consistent with previous studies, we classified mice with depression and anxiety-like behaviors induced by CSDS as susceptible and those without depression but anxiety behaviors after CSDS as resilient animals (Supplementary material A–E) [34, 35]. The raphe nuclei of three groups of mice (control, susceptible, and resilient) were obtained for mRNA-seq detection. The results of mRNA-seq yielded 242 differentially expressed genes between susceptible and control mice and 545 between susceptible and resilient mice (Fig. 1B). By taking the intersection of two groups of differentially expressed genes, we obtained 51 differentially expressed genes. There is reason to believe that these 51 genes are involved in depression and anxiety behaviors induced by CSDS (Fig. 1C). To determine the biological implications of this data set, Gene Ontology (GO) analysis was performed to determine the gene and gene product enrichment in the following categories: molecular function (MF), cellular component (CC), and biological process (BP). Among BP terms, sequence-specific DNA binding was identified as a significantly enriched network. In the CC category, the synapse network was significantly enriched. Among the MF terms, the most significantly enriched function was the regulation of NMDA receptor activity (Fig. 1D). Simultaneously, p11 was significantly reduced in the DRN of susceptible mice, and its expression correlated positively with the social index (Fig. 1E–H). Thus, the decrease in p11 expression in the DRN might be related to the depression- and anxiety-like phenotypes of CSDS mice.

### Knockdown of p11 in DDRN-induceddepression-like behaviors

**Fig. 2:**
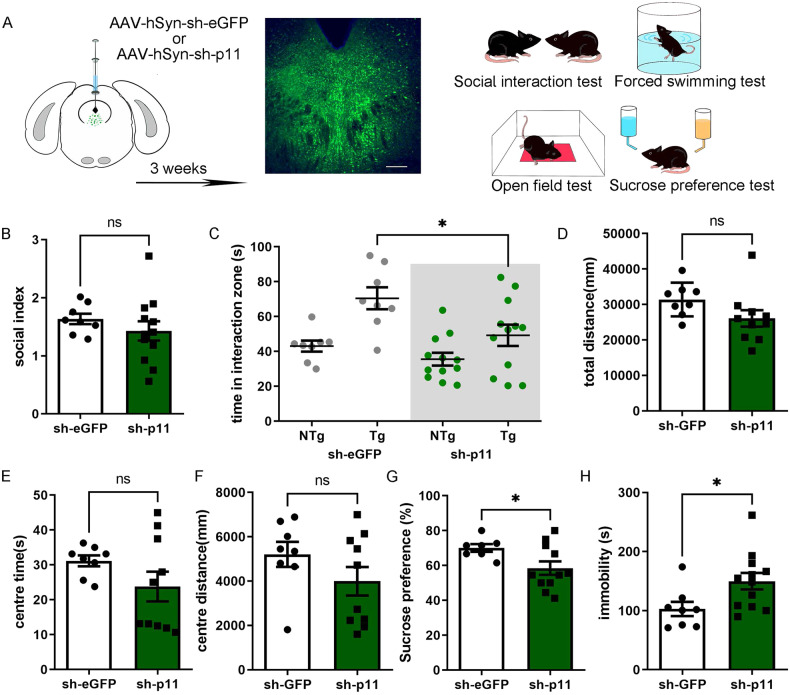
Knockdown of p11 in DRN induced depression-like behaviors. **A** Experimental paradigm for AAV-hSyn-sh-eGFP and AAV-hSyn-sh-p11 injection (left) and representative picture of injection site verification (middle) and the experimental timeline (right) for social interaction test (SIT), open field test (OFT), sucrose preference test (SPT) and forced swimming test (FST). **B** Social index in social interaction test of sh-p11 and sh-eGFP group. (sh-eGFP *n* = 8, sh-p11 *n* = 12, bars represent mean ± s.e.m. Two-tailed unpaired Students *t* test, *p* = 0.3574 *t* = 0.9445). **C** Time spent in the interaction zone in social interaction test of sh-p11 and sh-eGFP group when a target mice (Tg) or no target mice (NTg) in the social zone. (sh-eGFP NTg *n* = 8, sh-eGFP Tg *n* = 8, sh-p11 NTg *n* = 12 sh-p11 Tg *n* = 12, bars represent mean ± s.e.m. sh-eGFP Tg vs. sh-p11 Tg, Two-tailed unpaired Students *t* test, *p* = 0.0319 *t* = 2.326). **D** Total distance in the open field test of sh-p11 and sh-eGFP group. (sh-eGFP *n* = 8, sh-p11 *n* = 12, bars represent mean ± s.e.m. Two-tailed unpaired Students *t* test, *p* = 0.0972 *t* = 1.762). **E** Center time in the open field test of sh-p11 and sh-eGFP group. (sh-eGFP *n* = 8, sh-p11 *n* = 12, bars represent mean ± s.e.m. Two-tailed unpaired Students *t* test, *p* = 0.1587 *t* = 1.478). **F** Center distance in the open field test of sh-p11 and sh-eGFP group. (sh-eGFP *n* = 8, sh-p11 *n* = 12, bars represent mean ± s.e.m. Two-tailed unpaired Students *t* test, *p* = 0.1866 *t* = 1.380). **G** Percentage of sucrose preference in the sucrose preference test of Ctrl/Res/Sus mice. of sh-p11 and sh-eGFP group. (sh-eGFP *n* = 7, sh-p11 *n* = 11, bars represent mean ± s.e.m. Two-tailed unpaired Students *t* test, *p* = 0.0409 *t* = 2.223). **H** Immobility time in in forced swimming test of sh-p11 and sh-eGFP group. (sh-eGFP *n* = 8, sh-p11 *n* = 12, bars represent mean ± s.e.m. Two-tailed unpaired Students *t* test, *p* = 0.0296 *t* = 2.362).

To first determine whether the region-specific knockdown of p11 in DRN induces a similar effect as the constitutive knockdown of p11 in mice (p11-KO), we employed RNA interference to knockdown p11 in the DRN and observed the behavioral phenotypes. An adeno-associated virus (AAV)-delivered short hairpin RNA targeting p11 (AAV-hsyn-eGFP-miR30-p11.shRNA) and its control AAV (AAV-hsyn-eGFP-miR30-control.shRNA) were injected into the DRN of mice from two groups (sh-p11 and sh-eGFP, respectively), as shown in Fig. 2A. After verifying the knockout efficiency of the virus (Supplementary material F, G), the behavioral results suggested that specific knockdown of p11 in the DRN induced decreased social interaction, evidenced by lesser time spent in the interaction zone (Fig. 2C), although there was no significant difference in the social interaction index (Fig. 2B). Moreover, the knockdown of p11 in DRN induced behavioral despair and anhedonia (reduction in reward-related behaviors), indicated by decreased sucrose preference rate in the sucrose preference test (Fig. 2G) and increased immobility in the FST (Fig. 2H), respectively. However, in the open field test, p11-knocked-down mice showed no difference in the central time and the central distance compared to the control mice (Fig. 2D–F). These results suggested that the knockdown of p11 in DRN could induce depression-like behavior but not anxiety-like behavior in mice.

### p11 is mainly expressed in serotonergic neurons of DRN

**Fig. 3:**
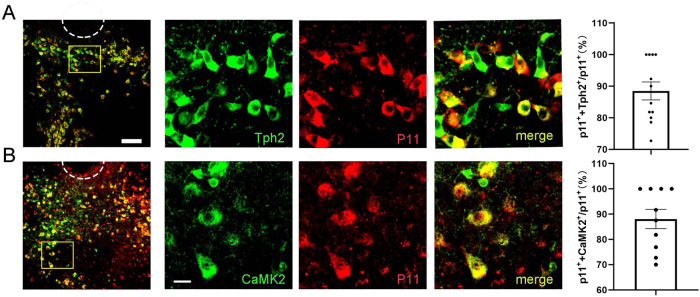
p11 is mainly expressed in serotonergic neurons of DRN. **A** Images showing the co-localization of p11 (red) with Tph2 (green, a marker of 5-HT neurons) in medial part of the DRN neurons. Large image: bar = 100 μm; Small image: bar = 10 μm. And the percentage of p11+ neurons expressing Tph2 in DRN (*n* slice = 12, *n* mice = 3). **B** Images showing the co-localization of p11 (red) with CaMK2 (green, a marker of glutmate neurons) in medial part of the DRN neurons. Large image: bar = 100 μm; Small image: bar = 10 μm. And the percentage of p11+ neurons expressing CaMK2 in DRN (*n* slice = 10, *n* mice = 3).

The DRN contains different types of neurons. A previous study reported that serotonergic neurons are distributed in the middle of the DRN while GABAergic neurons are distributed on both sides of the DRN [31]. To further confirm the cell types of p11-positive cells, we performed double-staining with different neuron markers and p11. We hypothesized that p11-positive neurons may have a specific cell type. 88.49 ± 2.847% of p11-positive neurons were serotonergic neurons (Fig. 3A), and 88.06 ± 3.812% of p11-positive neurons were glutamatergic neurons (Fig. 3B). This result suggests that p11-positive cells are most likely to be a class of serotonergic neurons that express excitatory transmitters.

### p11 overexpression in 5-HTDRN ameliorates stress-induced depression-like behavior

**Fig. 4:**
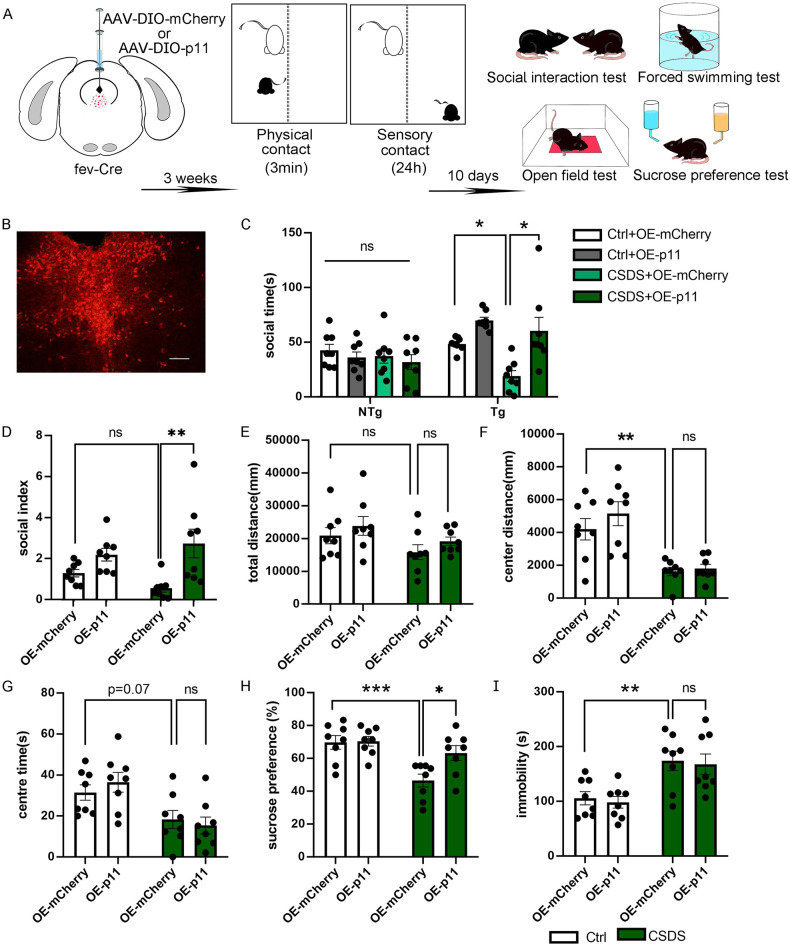
p11 overexpression in 5-HT^DRN^ ameliorates stress-induced depression-like behavior. **A** Experimental paradigm for AAV-DIO-mCherry and AAV-DIO-p11 injection (left) and experimental timeline for CSDS modeling (middle) and social interaction test (SIT), open field test (OFT), sucrose preference test (SPT) and forced swimming test (FST). **B** representative picture of injection site verification. **C** Time spent in the interaction zone in social interaction test of OE-mCherry and OE-p11 group when a target mice (Tg) or no target mice (NTg) in the social zone. (*n* = 8, bars represent mean ± s.e.m. Ordinary one-way ANOVA followed by Tukey’s post-hoc test, Tg: Ctrl-OE-mCherry vs. CSDS + OE-mCherry, adjusted *p* = 0.036, CSDS + OE-mCherry vs. CSDS + OE-p11, adjusted *p* = 0.0463). **D** Social index in social interaction test of Ctrl+OE-mCherry/ Ctrl+OE-p11/CSDS + OE-mCherry/CSDS + OE-p11 group. (*n* = 8, bars represent mean ± s.e.m. two-way ANOVA followed by Tukey’s post-hoc test, Ctrl+OE-mCherry vs CSDS + OE-mCherry, adjusted *p* = 0.3742, CSDS + OE-mCherry vs CSDS + OE-p11, adjusted *p* = 0.0014). **E** Total distance in the open field test of Ctrl+OE-mCherry/Ctrl+OE-p11/CSDS + OE-mCherry/CSDS + OE-p11 group. (*n* = 8, bars represent mean ± s.e.m. two-way ANOVA followed by Tukey’s post-hoc test, Ctrl+OE-mCherry vs CSDS + OE-mCherry, adjusted *p* = 0.2969, CSDS + OE-mCherry vs CSDS + OE-p11, adjusted *p* = 0.5315). **F** Center distance in the open field test of Ctrl+OE-mCherry/Ctrl+OE-p11/CSDS + OE-mCherry/CSDS + OE-p11 group. (*n* = 8, bars represent mean ± s.e.m. Ordinary one-way ANOVA by Tukey’s post-hoc test, Ctrl+OE-mCherry vs CSDS + OE-mCherry, adjusted *p* = 0.9630, CSDS + OE-mCherry vs CSDS + OE-p11, adjusted *p* = 0.008). **G** Center time in the open field test of Ctrl+OE-mCherry/Ctrl+OE-p11/CSDS + OE-mCherry/CSDS + OE-p11 group. (*n* = 8, bars represent mean ± s.e.m. two-way ANOVA followed by Tukey’s post-hoc test, Ctrl+OE-mCherry vs CSDS + OE-mCherry, adjusted *p* = 0.0771, CSDS + OE-mCherry vs CSDS + OE-p11, adjusted *p* = 0.8643). **H** Percentage of sucrose preference in the sucrose preference test of Ctrl+OE-mCherry/ Ctrl+OE-p11/CSDS + OE-mCherry/CSDS + OE-p11 group. (*n* = 8, bars represent mean ± s.e.m. two-way ANOVA followed by Tukey’s post-hoc test, Ctrl+OE-mCherry vs CSDS + OE-mCherry, adjusted *p* = 0.0005, CSDS + OE-mCherry vs CSDS + OE-p11, adjusted *p* = 0.0110). **I** Immobility time in the forced swimming test of Ctrl+OE-mCherry/Ctrl+OE-p11/CSDS + OE-mCherry/CSDS + OE-p11 group. (*n* = 8, bars represent mean ± s.e.m. two-way ANOVA followed by Tukey’s post-hoc test, Ctrl+OE-mCherry vs CSDS + OE-mCherry, adjusted *p* = 0.0072, CSDS + OE-mCherry vs CSDS + OE-p11, adjusted *p* = 0.9504).

As mentioned above, p11 was mainly expressed in serotonergic neurons and CSDS induced a significant reduction in its levels. Therefore, we sought to determine whether p11 in 5-HT^DRN^ was involved in stress-induced depression-like behaviors. To specifically overexpress p11 in 5-HT^DRN^, we injected Cre-dependent viruses into the DRN of fev-Cre mice [36], Previous research has demonstrated that the majority of neurons labeled in the DRN of fev-Cre mice exhibit co-expression of VGLUT3 and serotonergic markers [37]. These neurons in the DRN are believed to play a role in the modulation of aversive memory [38] and anhedonia-like behavior [39]. The spatial pattern of viral transduction corresponds closely with the anatomical distribution of p11-expressing neurons (Fig. 4B). Firstly, we compared the Ctrl+OE-mCherry group with the CSDS + OE-mCherry group to investigate whether brain stereotactic injection would impact the normal behavior of mice and whether it would influence the depressive behavior induced by CSDS. The findings revealed that mice overexpressing mCherry exhibited a reduction in social interaction time following CSDS stimulation (Fig. 4C), decreased central distance (Fig. 4F) and sucrose preference (Fig. 4H) in the open field test, and increased immobility time in the forced swimming test (Fig. 4I). These results suggest that viral injection does not affect the behavior of the mice or the impact of CSDS on their behavior. Secondly, we compared the CSDS + OE-mCherry group with the CSDS + OE-p11 group to assess the effects of p11 overexpression on social and hedonic behaviors induced by CSDS. The results demonstrated that after p11 overexpression, social interaction time increased (Fig. 4C), central distance in the open field test remained unchanged (Fig. 4F), sucrose preference increased (Fig. 4H), and immobility time in the forced swimming test did not differ significantly (Fig. 4I). These findings indicate that overexpression of p11 in serotonergic neurons can reverse the social and hedonic behavioral alterations induced by CSDS.

### p11 regulates membrane trafficking of glutamate receptors

**Fig. 5:**
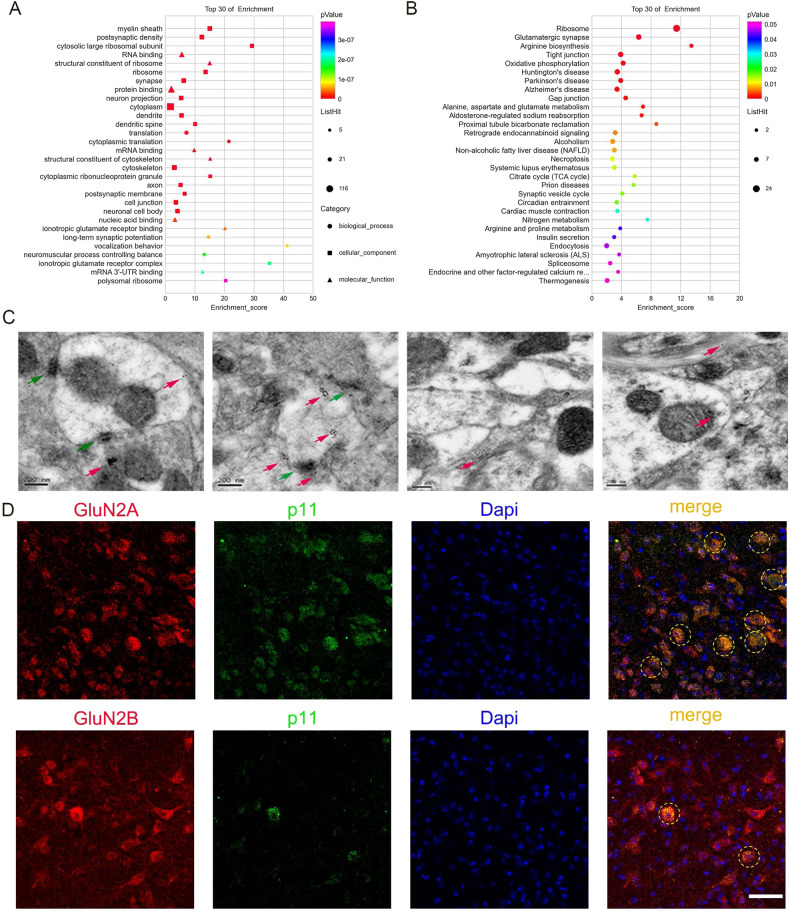
p11 located in synapses and was involved in regulating synaptic function. **A**, **B** Top 30 enriched (normalized enrichment score, NES > 0; false-discovery rate, FDR < 0.25) and de-enriched (NES < 0, FDR < 0.25) gene sets in the CSDS DRN samples revealed by gene set enrichment analysis (GSEA). GO-BP: gene ontology – biological process, GO-CC: gene ontology – cellular component, GO-MF: gene ontology – molecular function, KEGG: the Kyoto Encyclopedia of Genes and Genomes signaling pathway. **C** Representative pictures of immunoelectron microscopy. p11 in presynaptic, postsynaptic, axon, and cytoplasmic or cytoplasmic (left to right). Red arrow (labeled P11), green arrow (postsynaptic compact). **D** left: representative graph of immunofluorescence staining of GluN2A(red, up)/GluN2B(red, down), middle representative graph of immunofluorescence staining of p11(green) and p11(blue). co-localization of GluN2A(red, up)/GluN2B(red, down) p11 (green) and dapi(blue). scale bar 50 μM.

**Fig. 6:**
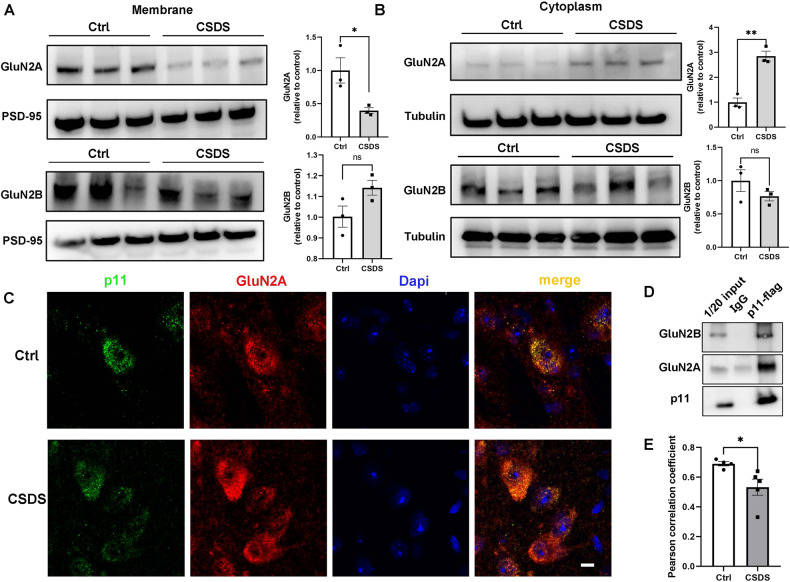
p11 involved in the expression of GluN2A on cell membrane. GluN2A (up) and GluN2B (down) protein level were assessed in the membranc (**A**-left) and cytoplasm (**B**-left) of ctrl mice and CSDS mice via Western blot. Comparison of grayscale value of GluN2A (up) and GluN2B (down) protein immunoblotting in the membranc (**A**-right) (GluN2A Ctrl *n* sample = 3, *n* mice = 9, CSDS *n* sample = 3, *n* mice = 9, bars represent mean ± s.e.m. Two-tailed unpaired Students *t* test, *p* = 0.0359 *t* = 3.110). (GluN2B Ctrl *n* sample = 3, *n* mice = 9, CSDS *n* sample = 3, *n* mice = 9, bars represent mean ± s.e.m. Two-tailed unpaired Students *t* test, *p* = 0.0894 *t* = 2.233), Comparison of grayscale value of GluN2A (up) and GluN2B (down) protein immunoblotting in the cytoplasm (B-right) (GluN2A Ctrl *n* sample = 3, *n* mice = 9, CSDS *n* sample = 3, *n* mice = 9, bars represent mean ± s.e.m. Two-tailed unpaired Students *t* test, *p* = 0.0021 *t* = 7.100). (GluN2B Ctrl *n* sample = 3, *n* mice = 9, CSDS *n* sample = 3, *n* mice = 9, bars represent mean ± s.e.m. Two-tailed unpaired Students *t* test, *p* = 0.2589 *t* = 1.315). **C** left: representative graph of immunofluorescence staining of p11(green), middle: representative graph of immunofluorescence staining of GluN2A (red)/dapi(blue), right: co-localization of p11 (green) with GluN2A (red)/dapi(blue). Ctrl-up, CSDS-down. scale bar 5 μM. **D** Co-immunoprecipitation (Co-IP) was performed to verify protein interaction between p11 and GluN2B (up)/ GluN2A (down). **E** Pearson correlation coefficient of p11 and GluN2A of Ctrl mice and CSDS mice(Ctrl *n* = 4, CSDS *n* = 5, bars represent mean ± s.e.m. Two-tailed unpaired Students *t* test, *p* = 0.0404 *t* = 2.510).

Then, we dissected DRN to screen for potential proteins that interacted with p11 by employing co-immunoprecipitation coupled with mass spectrometry (IP-MS). A total of 234 proteins could potentially interact with p11(Extended data), it contains Annexin II, a putative p11 interacting protein. GO and KEGG analysis showed that these proteins were mainly involved in the glutamate receptor signaling pathway, structural constituent of the synapse, and regulation of the synaptic vesicle cycle (Fig. 5A, B). To verify the results, we used colloidal gold particles to label p11 and observed its localization in neurons by immunoelectron microscopy. p11 was mostly found as aggregates and was widely expressed in presynaptic and postsynaptic membranes (Fig. 5C). Thus, p11 may regulate neural activity through synaptic modulation, consistent with existing literature, which indicates that p11 interacts with several ion channels and receptors and regulates their cellular localization and function [40–42]. Our Co-IP results showed that p11 interacted with both GluN2A and GluN2B (Fig. 6D) but co-localized more with GluN2A (Fig. 5D). In the CSDS animal model, we also found that after CSDS, GluN2A expression decreased in the cell membrane and increased in the cytoplasm (Fig. 6A, B), while its co-localization with p11 reduced markedly (Fig. 6C–E). Finally, these results suggested that p11 regulated the expression of GluN2A in the cell membrane, thus may influencing the activity of 5-HT^DRN^.

## Discussion

In this study, we identified the key role of p11 in the excitability of raphe serotonergic neurons and the mediation of depression-like behaviors. CSDS can significantly downregulate the protein and mRNA levels of p11 in DRN, thereby simultaneously inducing a depression-like phenotype. Knockdown of p11 in DRN caused depression-like phenotypes, which are similar to CSDS. Interestingly, p11 is predominantly expressed in serotonergic neurons in DRN. Furthermore, overexpression of p11 in serotonergic neurons of DRN can significantly relieve CSDS-induced depression-like behaviors. The most parsimonious explanation for our data is that p11 acts as a regulator of the surface expression of GluN2A in DRN, so its reduction might mediate CSDS-induced depression-like phenotypes by inhibiting the excitability of serotonergic neurons in DRN.

Based on the first study that showed depression-like behaviors due to conditional knockout of p11 [7], several subsequent reports have suggested that region- and cell-specific knockout of p11 in serotonergic projection regions can induce depression-like behaviors, such as in cortex [8], hippocampus [18], and NAc [8]. The effects were initially thought to be involved with the regulation of 5-HT receptor trafficking by p11, including 5-HT1B, 5-HT1D, and 5-HT4 [8, 16, 41]. However, the function of p11 associated with its high expression in DRN remains obscure (Fig. 3) [17]. In line with the study’s focus on the serotonergic projection regions, the knockdown of p11 in DRN, the region where serotonergic neurons are located, also resulted in depression-like phenotypes. However, the effect might be related to the trafficking of GluN2A, a subunit of the NMDA receptor to the surface of serotonergic neurons. 5-HT^DRN^ neurons receive glutamate excitatory inputs from several brain areas, as well as from interneurons within the same nucleus [43, 44]. The excitatory/inhibitory input balance governs the excitability of serotonergic neurons [45]. Therefore, according to the present results, we hypothesize that the knockdown of p11 reduces the surface expression of GluN2A decreases excitatory inputs, and subsequently lowers the excitability of serotonergic neurons which underlie the mechanism of depression.

In addition to the region- and cell-specific knockout of p11 in serotonergic projection regions inducing depression-like behaviors, lower mRNA and protein levels of p11 have been also found in the anterior cingulate cortex and the ventral striatum of depressed patients [9, 21] and in an animal model of depression [7, 46]. Although scientists have reported a high expression of p11 in DRN [20], few studies have focused on whether chronic stress, a stable risk factor for depression would influence the level of p11 in DRN. Our data showed that CSDS can induce a decrease in mRNA and protein levels of p11 in the DRN, especially in serotonergic neurons, and simultaneously result in depression-like phenotypes, giving more cues for the notion that p11 in the DRN is strongly associated with stress-induced depression.

As mentioned above, scientists first discovered that p11 controls depression-like behaviors through trafficking to the membrane in serotonergic receptors and modulation of synaptic transmission or neuronal excitability in specific types of neurons and specific brain regions, however, growing evidence suggests that in addition to 5-HT receptors, p11 also regulates non-serotonergic receptor surface expression, such as mGluR5, and ion channels [12, 47]. Recently, the presence of 5-HT and non-5-HT neurons was reported in the DRN. The latter include glutamatergic, GABAergic, and dopaminergic neurons [48–50]. The present study also confirmed the notion and verified that p11 is predominantly expressed in serotonergic neurons in DRN. Many studies also support the notion that 5-HT^DRN^ positively encodes a wide range of reward signals during anticipatory and consummatory phases of reward responses, suggesting that 5-HT neurons and the 5-HT signaling system mediate reward-related behaviors [5, 51]. In this study, overexpression of p11 in 5-HT^DRN^ neurons was found to be sufficient to reverse depression-like behavior caused by CSDS. Therefore, our results provide the possibility that the expression of p11 is related to the maintenance of 5-HT neuronal excitability in DRN and confirm the presence of p11 in neurons as an important molecular and cellular determinant of depression.

Several studies have shown that p11 is a multifunctional protein that forms a heterotetramer complex with Annexin A2 [17, 52–54], especially on the cell membrane. The heterotetrameric p11/AnxA2 complex traffics mGluR5 to the cell surface [12], but its role in the regulation of serotonin receptors’ surface expression has not been studied. In the present study, we also detected Annexin A2, GluN2A, and GluN2B in co-precipitated proteins with anti-p11 by Co-IP coupled MS. In GO and KEGG analyses for the co-precipitated proteins interacting with p11, we found that most proteins interacting with p11 may be involved in the construction of postsynaptic components, and their functions include protein binding, regulation of glutamate synapses, and synaptic vesicle cycling. Immunoelectron microscopy results showed that p11 had abundant pre- and post-synaptic distributions. As mentioned above, we believe that p11 is involved in the regulation of postsynaptic function by trafficking certain receptors to the cell membrane, especially in serotonergic neurons. NMDA receptors play crucial roles in excitatory synaptic transmission [55]. Further, WB analysis confirmed our hypothesis that GluN2A decreased on the membrane and increased in the cytoplasm in DRN, instead of GluN2B after exposure to CSDS. The activation of GluN2A receptors exerts pro-survival effects, while GluN2B receptors, which are mainly segregated to extrasynaptic sites, exhibit deleterious effects [56–58]. Functional restoration of GluN2A can play a neuroprotective role in improving anxiety-like and depression-like behaviors in AD mice [59]. Our results also suggest that GluN2A and GluN2B in DRN play different roles in regulating depression. Therefore, p11 in DRN may regulate synaptic function by altering the membrane distribution of GluN2A, thus influencing neuronal functions.

In the present study, we showed that knockdown or overexpression of p11 in DRN or serotonergic neurons can cause changes in depression behavior. Its possible mechanism may involve the interaction of p11 with GluN2A and the regulation of the GluN2A receptors’ surface expression. Thus, p11 may regulate the surface expression of GluN2A in the DRN. Our results provide more evidence for the regulation of non-serotonergic receptors by p11 in different brain regions.

## Conclusion

Herein, we provided evidence that p11 reduction in DRN neurons in a CSDS-induced depression model was closely related to the inhibition of serotonergic neurons in DRN and depression-like behavior. Overexpression of p11 in serotonergic neurons in DRN reversed depressive behavior, and the underlying molecular mechanism may regulate membrane trafficking of GluN2A.

### Supplementary information

Supplemental material

## Data Availability

Data for this study will be made available in a public archive following publication of this study. In the interim, data are available upon request.
