# Supplementary material for: Reduction of p11 in dorsal raphe nucleus serotonergic neurons mediates depression-like behaviors
Source: Transl Psychiatry. 2023 Nov 22;13:359. doi: 10.1038/s41398-023-02664-3 (PMC10665321; doi:10.1038/s41398-023-02664-3)
Supplement: Supplementary file 1 — Supplemental material [file 41398_2023_2664_MOESM1_ESM.pdf]

Supplementary materials

Supplement 1 CSDS induced depression and anxiety-like behaviors in susceptible mice and anxiety-like behaviors in resilient mice

Supplement 2 Extended data of all proteins potentially interacted with p11

Supplement 1 CSDS induced depression and anxiety-like behaviors in susceptible mice and anxiety-like behaviors in resilient mice

A. Ratio of social interaction in the social interaction test of Ctrl/Res/Sus mice. (Ctrl n=10, Res n=10, Sus n=10, bars represent mean ± s.e.m., ordinary one-way ANOVA followed by Tukey's post-hoc test, $F_{2/27} = 1.299$ Ctrl vs. Res adjust-p = 0.8976, Ctrl vs. Sus adjust-p < 0.0001, Res vs. Sus adjust-p < 0.0001)

B. Total distance in the open field test of Ctrl/Res/Sus mice. (Ctrl n=10, Res n=10, Sus n=10, bars represent mean ± s.e.m., ordinary one-way ANOVA followed by Tukey's post-hoc test, $F_{2/27} = 1.736$ Ctrl vs. Res adjust-p = 0.7353, Ctrl vs. Sus adjust-p =0.2645, Res vs. Sus adjust-p =0.6789)

C. Centre distance in the open field test of Ctrl/Res/Sus mice. (Ctrl n=10, Res n=10, Sus n=10, bars represent mean ± s.e.m., ordinary one-way ANOVA followed by Tukey's post-hoc test, $F_{2/27} = 2.028$ Ctrl vs. Res adjust-p = 0.0334, Ctrl vs. Sus adjust-p =0.0087, Res vs. Sus adjust-p =0.8371)

D. Percentage of sucrose preference in the sucrose preference test of Ctrl/Res/Sus mice. (Ctrl n=10, Res n=10, Sus n=10, bars represent mean ± s.e.m., ordinary one-way ANOVA followed by Tukey's post-hoc test, $F_{2/27} = 1.297$ Ctrl vs. Res adjust-p = 0.6473, Ctrl vs. Sus adjust-p =0.0041, Res vs. Sus adjust-p =0.0349)

E. Immobility time in in forced swimming test of Ctrl/Res/Sus mice. (Ctrl n=10, Res n=10, Sus n=10, bars represent mean ± s.e.m., ordinary one-way ANOVA followed by Tukey's post-hoc test, $F_{2/27} = 13.91$ Ctrl vs. Res adjust-p = 0.7979, Ctrl vs. Sus adjust-p =0.0001, Res vs. Sus adjust-p =0.0007.

F-G. p11 protein level of sh-eGFP mice and sh-p11 mice were assessed via Western blot. (sh-eGFP, n=3, sh-p11, n=3, bars represent mean ± s.e.m. Two-tailed unpaired Students t-test, p=0.0008).

Note: In all panels, $*p < 0.05$, $**p < 0.01$, $***p < 0.001$, ns $p > 0.05$.

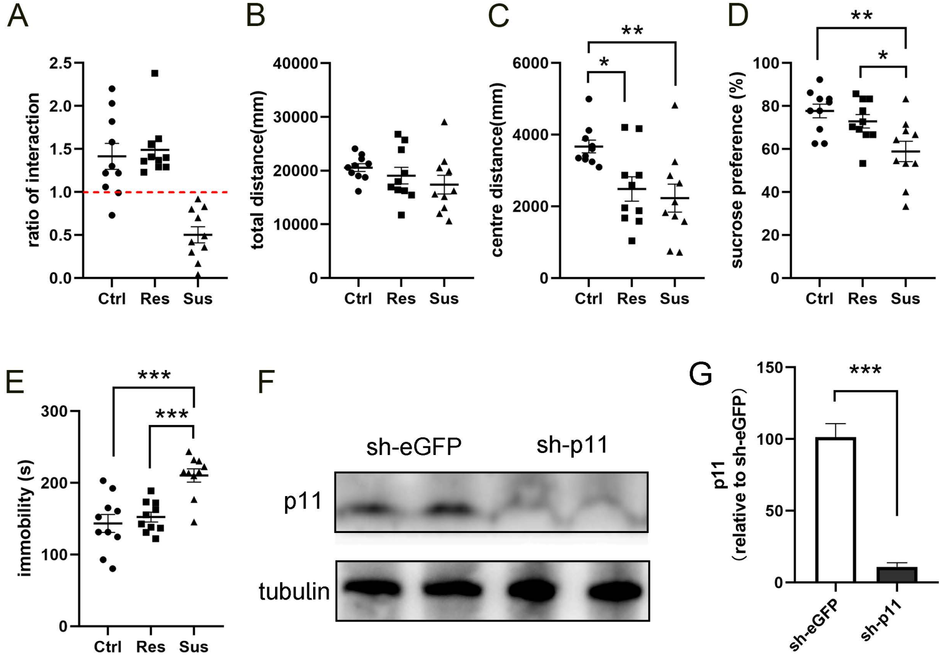
A
ratio of interaction
2.5
2.0
1.5
1.0
0.5
0.0
Ctrl
Res
Sus
B
total distance(mm)
40000
30000
20000
10000
0
Ctrl
Res
Sus
C
centre distance(mm)
6000
4000
2000
0
*
**
Ctrl
Res
Sus
D
sucrose preference (%)
100
80
60
40
20
0
**
*
Ctrl
Res
Sus
E
immobility (s)
300
200
100
0
***
***
Ctrl
Res
Sus
F
sh-eGFP
sh-p11
p11
tubulin
G
p11
(relative to sh-eGFP)
150
100
50
0
***
sh-eGFP
sh-p11

Master Protein Accession	Entry name	Coverage [%]	# Peptides	# PSMs	# Unique Peptides
Q9CWF2	TBB2B_MOUSE	45	14	22	1
D3YXK2	SAFB1_MOUSE	25	21	23	21
Q91VM5	RMXL1_MOUSE	37	17	19	3
Q61879	MYH10_MOUSE	19	29	31	20
Q8R0S2	IQEC1_MOUSE	21	19	19	14
Q8VDD5	MYH9_MOUSE	11	19	20	10
Q8K2F8	LS14A_MOUSE	26	13	16	13
Q62095	DDX3Y_MOUSE	24	16	18	2
Q9ERD7	TBB3_MOUSE	30	10	14	3
Q9QWI6	SRCN1_MOUSE	18	17	17	17
Q569Z6	TR150_MOUSE	16	13	16	13
E9QAT4	SC16A_MOUSE	9	12	13	12
Q6PIC6	AT1A3_MOUSE	19	16	16	6
P08207	S10AA_MOUSE	34	11	11	5
P68368	TBA4A_MOUSE	31	12	14	4
P68369	TBA1A_MOUSE	29	11	13	3
Q7TSJ2	MAP6_MOUSE	20	14	14	14
Q6PCN7	HLTF_MOUSE	15	13	13	3
O88569	ROA2_MOUSE	39	10	11	10
O88532	ZFR_MOUSE	13	10	10	10
P16546	SPTN1_MOUSE	7	14	14	14
Q7TN98	CPEB4_MOUSE	20	10	10	10
Q3TJZ6	FA98A_MOUSE	23	7	7	6
Q62261	SPTB2_MOUSE	7	13	13	13
F6SEU4	SYGP1_MOUSE	9	8	8	8
D3YZU1	SHAN1_MOUSE	9	11	11	11
Q99LF4	RTCB_MOUSE	15	8	9	8
Q3UTQ8	CDKL5_MOUSE	10	7	7	7
Q811P8	RHG32_MOUSE	5	8	8	8
Q60865	CAPR1_MOUSE	15	10	10	10
O88623	UBP2_MOUSE	10	7	7	7
Q9Z2K1	K1C16_MOUSE	14	6	6	2
Q8K310	MATR3_MOUSE	9	6	7	6
P28652	KCC2B_MOUSE	10	4	5	1
Q9CX86	ROA0_MOUSE	26	5	5	5
Q8C3F2	F120C_MOUSE	7	5	5	5
Q99MI1	RB6I2_MOUSE	4	5	5	1
O88735	MAP7_MOUSE	9	7	7	7
Q62108	DLG4_MOUSE	11	6	6	6
Q9R0H5	K2C71_MOUSE	6	4	6	1
Q8BFZ3	ACTBL_MOUSE	19	6	7	1
Q501J6	DDX17_MOUSE	11	6	6	3
P47911	RL6_MOUSE	17	5	5	5
P29341	PABP1_MOUSE	13	7	7	7
P01864	GCAB_MOUSE	17	4	5	4
Q61584	FXR1_MOUSE	10	5	5	4
O35927	CTND2_MOUSE	7	5	5	5

P19246	NFH_MOUSE	5	4	4	3
P01756	HVM12_MOUSE	43	3	3	1
P27659	RL3_MOUSE	12	4	4	4
Q8BH59	CMC1_MOUSE	6	4	4	4
Q9QYX7	PCLO_MOUSE	1	5	5	5
Q8K4G5	ABLM1_MOUSE	6	3	3	3
Q9R0I7	YLPM1_MOUSE	4	4	4	4
P09405	NUCL_MOUSE	7	4	4	4
P51881	ADT2_MOUSE	12	4	5	4
P42669	PURA_MOUSE	24	3	3	3
Q9EPQ8	TCF20_MOUSE	3	4	4	4
P18872	GNAO_MOUSE	11	3	3	3
Q9WVR4	FXR2_MOUSE	5	3	3	2
Q8C015	PAK5_MOUSE	8	4	4	4
Q8K019	BCLF1_MOUSE	5	5	5	5
Q64475	H2B1B_MOUSE	28	3	3	3
Q69ZX8	ABLM3_MOUSE	6	3	3	3
P62270	RS18_MOUSE	21	4	4	4
P25444	RS2_MOUSE	10	3	3	3
Q7TQH0	ATX2L_MOUSE	4	3	3	3
P39447	ZO1_MOUSE	3	4	4	4
A2AJA9	AJM1_MOUSE	4	3	3	3
Q8BP71	RFOX2_MOUSE	11	3	3	3
Q99N95	RM03_MOUSE	12	3	3	3
P61255	RL26_MOUSE	18	3	3	3
Q9DBR0	AKAP8_MOUSE	6	3	3	3
Q9D6M3	GHC1_MOUSE	7	3	3	3
Q99020	ROAA_MOUSE	8	2	2	1
Q80VD1	FA98B_MOUSE	6	2	2	1
P20029	BIP_MOUSE	5	2	2	1
O88935	SYN1_MOUSE	3	2	2	2
Q3UHB8	CC177_MOUSE	5	3	3	3
Q8BYI9	TENR_MOUSE	2	2	2	2
Q8K2M0	RM38_MOUSE	5	2	2	2
P47915	RL29_MOUSE	12	2	2	2
P01750	HVM06_MOUSE	27	3	3	2
E9Q557	DESP_MOUSE	1	3	3	3
O88737	BSN_MOUSE	1	2	2	2
Q922P9	GLYR1_MOUSE	6	2	2	2
P86048	RL10L_MOUSE	12	3	3	3
P16460	ASSY_MOUSE	5	2	2	2
Q5SYD0	MYO1D_MOUSE	3	2	2	2
O88291	ZN326_MOUSE	4	2	2	2
P35922	FMR1_MOUSE	4	2	2	1
P62918	RL8_MOUSE	14	3	3	3
A0A087WPF7	AUTS2_MOUSE	4	3	3	3
P61358	RL27_MOUSE	13	2	2	2
P18528	HVM57_MOUSE	19	1	1	1
Q3UTJ2	SRBS2_MOUSE	2	2	2	2
Q6PFD5	DLGP3_MOUSE	2	2	2	2

Q8CI61	BAG4_MOUSE	6	2	2	2
Q8CCS6	PABP2_MOUSE	15	1	1	1
P01872	IGHM_MOUSE	6	3	3	3
P56564	EAA1_MOUSE	4	1	1	1
P18531	HVM60_MOUSE	14	1	1	1
Q60598	SRC8_MOUSE	4	2	2	2
Q8K083	ZN536_MOUSE	2	1	1	1
O88207	CO5A1_MOUSE	2	1	1	1
Q8VDM6	HNRL1_MOUSE	3	2	2	2
P97350	PKP1_MOUSE	2	1	1	1
P35436	NMDE1_MOUSE	2	1	1	1
Q08189	TGM3_MOUSE	2	1	1	1
Q9CQC7	NDUB4_MOUSE	19	1	1	1
P97351	RS3A_MOUSE	5	1	1	1
Q9Z2X1	HNRPF_MOUSE	6	2	2	1
Q9CR57	RL14_MOUSE	6	1	1	1
Q8BMA3	CNKR3_MOUSE	4	1	1	1
Q9R1Z8	VINEX_MOUSE	1	1	1	1
Q9D067	MDM1_MOUSE	2	1	1	1
P62751	RL23A_MOUSE	15	2	2	2
P84244	H33_MOUSE	7	1	1	1
P67984	RL22_MOUSE	10	1	1	1
P03987	IGHG3_MOUSE	7	2	2	2
P47754	CAZA2_MOUSE	10	2	2	2
P43006	EAA2_MOUSE	2	1	1	1
P01635	KV5A3_MOUSE	11	1	1	1
P07901	HS90A_MOUSE	3	1	1	1
Q6ZWV7	RL35_MOUSE	19	2	2	2
P18525	HVM54_MOUSE	14	1	1	1
Q80Z38	SHAN2_MOUSE	1	1	1	1
P39053	DYN1_MOUSE	1	1	1	1
Q8C3Q5	SHSA7_MOUSE	2	1	1	1
Q9JKS5	HABP4_MOUSE	4	1	1	1
P62814	VATB2_MOUSE	3	1	1	1
P97379	G3BP2_MOUSE	3	1	1	1
Q9CY57	CHTOP_MOUSE	5	1	1	1
P01633	KV5A1_MOUSE	6	1	1	1
Q9Z2D6	MECP2_MOUSE	4	1	1	1
Q9DC70	NDUS7_MOUSE	4	1	1	1
Q9CXS4	CENPV_MOUSE	8	1	1	1
Q9Z204	HNRPC_MOUSE	7	2	2	2
P14106	C1QB_MOUSE	5	1	1	1
P01831	THY1_MOUSE	9	1	1	1
P35486	ODPA_MOUSE	5	2	2	2
O55125	NIPS1_MOUSE	5	1	1	1
Q9CR59	G45IP_MOUSE	4	1	1	1
Q8R1A4	DOCK7_MOUSE	1	1	1	1
P63085	MK01_MOUSE	3	1	1	1
Q9R0T8	IKKE_MOUSE	2	1	1	1

P62862	RS30_MOUSE	17	1	1	1
Q62420	SH3G2_MOUSE	3	1	1	1
P15105	GLNA_MOUSE	4	1	1	1
Q9D0E1	HNRPM_MOUSE	1	1	1	1
P59999	ARPC4_MOUSE	7	1	1	1
Q8K0U4	HS12A_MOUSE	2	1	1	1
Q9DB77	QCR2_MOUSE	4	1	1	1
Q8BIZ1	ANS1B_MOUSE	2	2	2	2
Q5DTT2	PSD1_MOUSE	1	1	1	1
P62717	RL18A_MOUSE	7	1	1	1
P52480	KPYM_MOUSE	2	1	1	1
P61264	STX1B_MOUSE	5	1	1	1
P01630	KV2A6_MOUSE	12	1	1	1
P05201	AATC_MOUSE	3	1	1	1
Q9WTL4	INSRR_MOUSE	1	1	1	1
Q01097	NMDE2_MOUSE	1	1	1	1
P56135	ATPK_MOUSE	14	1	1	1
P62852	RS25_MOUSE	8	1	1	1
Q91XM9	DLG2_MOUSE	2	1	1	1
P46737	BRCC3_MOUSE	3	1	1	1
P61963	DCAF7_MOUSE	4	1	1	1
P06328	HVM49_MOUSE	6	1	1	1
P83882	RL36A_MOUSE	8	1	1	1
Q4ACU6	SHAN3_MOUSE	1	1	1	1
Q9JJ80	RPF2_MOUSE	4	1	1	1
Q9QY06	MYO9B_MOUSE	0	1	1	1
P04940	KV6A6_MOUSE	7	1	1	1
Q8CH25	SLTM_MOUSE	3	3	3	3
P62900	RL31_MOUSE	7	1	1	1
Q61464	ZN638_MOUSE	1	1	1	1
Q64332	SYN2_MOUSE	6	2	2	2
P61514	RL37A_MOUSE	20	1	1	1
P84099	RL19_MOUSE	5	1	1	1
Q60759	GCDH_MOUSE	1	1	1	1
Q8C8R3	ANK2_MOUSE	0	1	1	1
P30999	CTND1_MOUSE	1	1	1	1
Q9Z110	P5CS_MOUSE	1	1	1	1
Q9QYR6	MAP1A_MOUSE	1	2	2	2
P07356	ANXA2_MOUSE	3	1	1	1
Q99JY9	ARP3_MOUSE	3	1	1	1
Q6EJB6	UT14B_MOUSE	1	1	1	1
P06327	HVM52_MOUSE	13	1	1	1
P12960	CNTN1_MOUSE	1	1	1	1
Q61361	PGCB_MOUSE	1	1	1	1
Q91VD9	NDUS1_MOUSE	1	1	1	1
Q99KI0	ACON_MOUSE	2	1	1	1
Q3UIL6	PKHA7_MOUSE	1	1	1	1
B1AZP2	DLGP4_MOUSE	1	1	1	1
P70372	ELAV1_MOUSE	2	1	1	1
P14148	RL7_MOUSE	2	1	1	1

Q9DBR1	XRN2_MOUSE	5	3	3	3
Q9CQL5	RM18_MOUSE	5	1	1	1
Q8K0S0	PHYIP_MOUSE	2	1	1	1
Q8K2B3	SDHA_MOUSE	2	1	1	1
P61164	ACTZ_MOUSE	2	1	1	1
P01819	HVM43_MOUSE	4	1	1	1
Q01097	NMDE2_MOUSE	2	2	1	1
P46096	SYT1_MOUSE	2	1	1	1
P61161	ARP2_MOUSE	2	1	1	1
Q07076	ANXA7_MOUSE	2	1	1	1
Q8CFI0	NED4L_MOUSE	1	1	1	1
P30275	KCRU_MOUSE	4	1	1	1
Q9CZ13	QCR1_MOUSE	3	1	1	1
Q64523	H2A2C_MOUSE	9	1	1	1
Q91WD5	NDUS2_MOUSE	4	1	1	1
Q8C008	DZAN1_MOUSE	2	1	1	1
Q8BFU2	H2A3_MOUSE	8	1	1	1
Q9Z0E0	NCDN_MOUSE	2	1	1	1
P26443	DHE3_MOUSE	3	1	1	1
Q80TE7	LRRC7_MOUSE	2	2	2	2
Q9D0M5	DYL2_MOUSE	12	1	1	1
P84104	SRSF3_MOUSE	13	1	1	1
E9Q735	UBE4A_MOUSE	4	1	1	1
Q9D3V1	DRC10_MOUSE	4	1	1	1
Q9EPJ9	ARFG1_MOUSE	2	1	1	1
Q80SW1	SAHH2_MOUSE	2	1	1	1
Q60722	ITF2_MOUSE	1	1	1	1
A6X935	ITIH4_MOUSE	3	1	1	1
P14869	RLA0_MOUSE	10	1	1	1
O09167	RL21_MOUSE	9	1	1	1
E9Q7X7	NRX2A_MOUSE	2	1	1	1
Q8CDP0	CBPC3_MOUSE	4	1	1	1
Q8BL65	ABLM2_MOUSE	2	1	1	1
Q8CGZ0	CHERP_MOUSE	1	1	1	1
Q8BZN4	NUAK2_MOUSE	3	1	1	1
P62874	GBB1_MOUSE	3	1	1	1
Q3UVX5	GRM5_MOUSE	2	1	1	1
